# Protective Effects of ω-3 PUFA in Anthracycline-Induced Cardiotoxicity: A Critical Review

**DOI:** 10.3390/ijms18122689

**Published:** 2017-12-12

**Authors:** Simona Serini, Renata Ottes Vasconcelos, Renata Nascimento Gomes, Gabriella Calviello

**Affiliations:** 1 Istituto di Patologia Generale, Università Cattolica del Sacro Cuore, Largo Francesco Vito, 1-00168 Roma, Italy; simona.serini@unicatt.it; 2 Center for Translational Research in Oncology (Laboratory of Medical Investigation #24), Department of Radiology and Oncology, School of Medicine of São Paulo University, Cancer Institute of São Paulo, São Paulo 01246-000, Brazil; renataottes@yahoo.com.br (R.O.V.); renataalex@usp.br (R.N.G.)

**Keywords:** anthracyclines, cardioprotection, cardiotoxicity, chemotherapy, ω-3 PUFA

## Abstract

It has been demonstrated that ω-3 polyunsaturated fatty acids (ω-3 PUFA) may exert a beneficial role as adjuvants in the prevention and treatment of many disorders, including cardiovascular diseases and cancer. Particularly, several in vitro and in vivo preclinical studies have shown the antitumor activity of ω-3 PUFA in different kinds of cancers, and several human studies have shown that ω-3 PUFA are able to decrease the risk of a series of cardiovascular diseases. Several mechanisms have been proposed to explain their pleiotropic beneficial effects. ω-3 PUFA have also been shown to prevent harmful side-effects (including cardiotoxicity and heart failure) induced by conventional and innovative anti-cancer drugs in both animals and patients. The available literature regarding the possible protective effects of ω-3 PUFA against anthracycline-induced cardiotoxicity, as well as the mechanisms involved, will be critically discussed herein. The study will analyze the critical role of different levels of ω-3 PUFA intake in determining the results of the combinatory studies with anthracyclines. Suggestions for future research will also be considered.

## 1. Introduction

ω-3 polyunsaturated fatty acids (PUFA) are dietary factors acknowledged for their ability to induce multiple beneficial effects [1,2,3,4]. The protection exerted by these fatty acids has been associated to their ability to prevent pathologies affecting various tissues and organs, by modulating a variety of cellular processes, molecular pathways and factors [5,6,7,8]. Initially, the cardiovascular (CV) area was identified as the site where an increased intake of ω-3 could preferentially carry out their beneficial action [3]. Only subsequently was it hypothesized that ω-3 PUFA could also protect against the development and progression of several kinds of cancers. Among the mechanisms proposed for their anticancer activity, some are similar to those involved in their CV protective effects, while others are more specifically involved in cancer cell growth and survival [9,10,11,12]. Particular attention has been focused on the possibility that these dietary factors may act as adjuvants of already existing antineoplastic drugs, either by increasing the sensitivity of cancer cells towards these drugs or by reducing their dangerous side-effects [13,14]. In the present review, a brief discussion will be firstly outlined on the possibility to prevent both CV diseases and cancer through an increased intake of these fatty acids, and the mechanisms involved. In particular, their combinations with antineoplastic agents will be considered. Moreover, the possibility that ω-3 PUFA may reduce the cardiotoxicity induced by chemotherapy—particularly with anthracyclines (ATC)—will be critically analyzed. This is a topic of great interest, being ATC still largely used in a variety of cancers, mainly in combination with conventional or innovative drugs [15].

## 2. ω-3 PUFA and Cardiovascular (CV) Diseases

CV diseases (CVDs) are chronic non-communicable diseases responsible for the highest morbidity and mortality worldwide and, according to the last report by the World Health Organization, they kill 17.7 million people each year [16]. Currently, diet and lifestyle are considered major risk factors for CVD [17,18]. On the other hand, intervention on both factors may help in the prevention of these diseases and their clinical events. In particular, it is acknowledged that dietary ω-3 PUFA are among the nutritional factors which beneficially affect the frequency and severity of CVD [3,19]. α-Linolenic acid (ALA 18:3ω-3), found in vegetables, is the precursor of the most effective long-chain PUFA (LC-PUFA) eicosapentaenoic acid (EPA 20:5ω-3) and docosahexaenoic acid (DHA 22:6ω-3), present at high levels in marine fatty fish and seafood [20,21,22]. A great number of studies have directly associated ω-3 dietary consumption to a series of beneficial effects at CV level, such as: prevention of arrhythmias [23], platelet aggregation [24], and arterial inflammatory responses [25]; normalization of plasma triacylglycerol [26] and blood pressure [27]; and improvement of vascular relaxation [27] and heart rate variability [28,29].

The specific mechanisms underlying the cardioprotective effects of ω-3 PUFA are not fully understood. It is believed that the ability of these dietary fatty acids to exert pleiotropic effects in cells and tissues is mainly related to the fact that they are incorporated in structural phospholipids of cellular membranes [30,31], and alter their physicochemical properties, such as permeability, deformability [32,33], and fluidity [3]. Consequently, cellular components and signaling pathways may be affected [34]. For instance, the relative refractory period of myocyte voltage-gated sodium channels may be prolonged, the voltage required for membrane depolarization increased, thus leading to anti-arrhythmic effects [35]. Moreover, the ω-3 modulatory action on l-type calcium channels may result in reduced cytosolic free Ca^2+^ and Ca^2+^ influx rate, which is believed to prevent the cytosolic calcium overload taking place during the ischemic insult [36]. The anti-inflammatory and anti-thrombotic effects of ω-3 PUFA have mainly been related to their metabolic conversion to oxygenated derivatives, which are broadly named oxylipins, and are highly bioactive factors acting at very low concentrations [37,38]. Following a stimulus, both the LC-ω-3 PUFA EPA and DHA or the ω-6 PUFA arachidonic acid (AA), are released from cell membranes and converted to oxylipins with similar structures, but often contrasting effects. There are three main pathways for these conversions, mediated by the enzymes cyclooxygenase (COX), lipoxygenase (LOX), and cytochrome P-450 (Cyt P450). As the tissue level of LC-ω-3 PUFA increases, they compete with AA for COX-2 and LOX enzymatic conversions, increasing the production of 3-series prostaglandins (PG), which are vasodilators and platelet aggregation inhibitors [39], and that of thromboxane A_3_ (TXA3) and leukotriene B_5_ (LTB5) which, compared to the analogous AA-derived compounds (PGE2 and LTB4), are weaker platelet aggregators and less strong inducers of inflammation [40]. Lastly, ω-3 PUFA give rise to resolvins, metabolic derivatives with powerful anti-inflammatory and pro-resolving effects [41]. At the CV level, the increased formation of these specific LC-ω-3 PUFA products are thought to reduce atherosclerotic plaque formation and induce their stabilization by decreasing the infiltration of inflammatory and immune cells [42]. The ability of ω-3 PUFA to inhibit the secretion of very low density lipoproteins (VLDL) from liver, as well as the conversions of VLDL to intermediate-density lipoprotein (IDL), and low density lipoproteins (LDL) has been related to their hypotriglyceridemic effect [43]. On the other hand, this effect has also been explained based on the ability of ω-3 fatty acids to increase β-oxidation, thus leading to the reduction of the fatty acid substrate for triglyceride synthesis [44,45,46].

## 3. ω-3 PUFA and Cancer

Several preclinical (in vitro and in vivo) studies and some human experimental studies have demonstrated the ability of ω-3 PUFA to decrease cancer cell proliferation, to promote cancer cell death and to inhibit neoangiogenesis in cancer [47,48,49]. Interestingly, these anticancer effects are induced by ω-3 PUFA only in cancer cells and not in normal cells [50]. Among the main mechanisms involved in the ω-3 PUFA antineoplastic action, there is the modulation of cell proliferation and survival, that is associated to their ability to affect the expression and/or function of genes, proteins (membrane carriers and receptors, intracellular components of signaling pathways) and lipid mediators (oxylipins) involved in these biological processes. One possibility that these compounds have in obtaining this result is linked to their ability to modify the chemical structure and physical properties of those specialized lipid microenvironments in plasma membranes (lipid rafts) [6,30,31,51,52], where multiple factors involved in signaling are concentrated and interact each other. The alterations induced by ω-3 PUFA in membranes can substantially modulate important signaling pathways for cancer development. As a result, they can reduce the expression/activity of the nuclear transcription factor-κB (NF-κB) in several kinds of cancer cells, thus inducing apoptosis [53,54,55,56]. ω-3 PUFA can also inhibit cancer cell growth by altering the levels of several factors involved in the progression of the cell cycle, such as cyclins, cyclin-dependent kinases and retinoblastoma protein [31]. Another mechanism through which ω-3 PUFA are thought to act is due to their chemical structure and susceptibility to peroxidation. It has been suggested that the incorporation of these PUFA in membranes and mitochondrial phospholipids can sensitize tumor cells to oxygen reactive species (ROS) [57,58]. Moreover, it is known that lipid peroxidation products may directly inhibit DNA synthesis, cell growth, and induce tumor cell death [59,60,61].

Furthermore, there is general agreement on the fact that the powerful anticancer effects exerted by ω-3 PUFA may be partly related to their metabolic conversion to oxylipins. These derivatives have been shown to influence key events in processes involved in the development of cancer, such as cell proliferation, survival and inflammation. We have already considered that, overall, some of the oxylipins derived from EPA and DHA metabolism exert less powerful pro-inflammatory actions than the analogous products originating from AA (i.e., PG), and that other LC-ω-3 PUFA specific products (i.e., resolvins) prevent inflammation and induce its resolution [41]. These anti-inflammatory properties are worth underlining, since inflammation has been considered to play a central role in the development of several cancers. Moreover, while AA-derived oxylipins usually promote cancer cell proliferation, those derived from ω-3 exert a clear anti-proliferative role [62]. The formation of AA-derived products is usually controlled, but excessive concentrations are produced in pathological conditions, such as cancer. By competing with AA for both the incorporation in membranes and oxidative metabolism, EPA and DHA have the potential to induce a decrease of the AA-derived products, and, thus, to reduce the molecular responses associated with AA metabolism [31,50].

## 4. Potential Adjuvant Role of ω-3 PUFA in Combination with Antineoplastic Drugs

The combined treatment of ω-3 PUFA with other already used anticancer chemotherapeutics represents the more possible application of these nutrients in cancer therapy [13]. To date, the potential adjuvant role of ω-3 PUFA has been investigated in combination with a series of conventional drugs in a wide range of cancers (for a comprehensive review, see [13]). The ability of these fatty acids to reduce the toxic side-effects of these drugs has been largely proven [63,64,65], and several results have also concurred to demonstrate their chemosensitizing effects, as well as their ability to prevent drug-resistance [66,67,68]. In a recent review that comprehensively analyzed all the results of the existing combinatory studies, we concluded that most of them supported a potential adjuvant-role for ω-3 PUFA [13]. The findings achieved in the two last years have further substantiated the results analyzed in our review. The new information was obtained either by using different cancer cell models, or by studying new molecular and cellular mechanisms for the previously investigated combinations, such as DHA combined with 5-fluorouracyl [69], or cisplatin [70], or docetaxel [71]. Overall, the suppression of cell survival pathways and induction of cell death are among the most important mechanisms to explain the chemosensitizing effects of these fatty acids in a variety of cancers [13]. The suppression of cancer cell stemness has also been recently reported [68], and multiple results have suggested that oxidative stress-induced cytotoxicity plays a central role [13,48,68], particularly if the drugs used in combination with ω-3 PUFA were themselves inducers of oxidative stress (such as ATC, or disulfiram) [68].

Some recent papers have also investigated the ability of ω-3 to sensitize cells to the action of new-generation single-targeted drugs. For instance, the combined treatment with LC-ω-3 PUFA and bortezomib synergistically induced apoptosis and increased the sensitivity of human myeloma cells to this drug [72]. DHA combined with trastuzumab, a specific inhibitor of the HER2 receptor, increased the efficacy of this drug in inhibiting the growth of Her2/neu positive breast cancer cells, by synergistically reducing the expression of the phosphorylated form of extracellular signal–regulated kinase (p-) and protein kinase B (p-AKT) [73]. Moreover, the combined treatment of DHA with everolimus or barasertib synergistically promoted ROS-dependent cytotoxicity in Jurkat acute lymphoblastic leukemia cells [65].

Human studies investigating the activity of ω-3 PUFA, such as chemosensitizers or suppressors of drug-resistance, are still scarce. In a phase II trial, Bougnoux et al. [74] reported that treatment with DHA, in combination with ATC-based chemotherapy, could improve the time to progression and overall survival in metastatic breast cancer patients, in particular of those able to incorporate high levels of DHA in plasma phospholipids. Accordingly, in patients with advanced non-small cell lung cancer [75], the addition of fish oil (FO) to standard chemotherapy with carboplatin/vinorelbine or carboplatin/gemcitabine proved to have the potential to enhance the efficacy of these treatments (increase in one-year survival index, response rate and clinical benefit). On the contrary, many human studies have reported that ω-3 PUFA may reduce some of the chemotherapy-induced harmful side-effects, including drug-induced cardiotoxicity (specifically ATC-induced, see Section 5), thus improving chemotherapy tolerance and prognosis [13,76,77]. 

## 5. Can Anthracyclines (ATC)-Induced Cardiotoxicity Be Prevented by ω-3 PUFA?

Almost all the results related to the potential of ω-3 PUFA in preventing cardiac events induced by chemotherapy refer to the treatments with the ATC antibiotics [78,79,80,81,82,83]. The possibility to prevent ATC-induced cardiotoxicity is a field of great interest, since this class of drugs is still the most extensively used in a variety of human solid and hematological cancers (e.g., breast cancer, sarcoma, lymphoma and pediatric leukemia) in combination with other categories of conventional chemotherapeutic agents or new-generation targeted drugs [15]. From a chemical point of view, these drugs are planar molecules consisting of a rigid hydrophobic tetracycline ring to which a daunosamine sugar is attached through a glycosidic bond. These compounds were introduced as chemiotherapic drugs for the first time in the 1960s [84]. Doxorubicin (DOX) and daunorubicin (DNR) are the two naturally occurring anthracyclines, derived from the bacterium *Streptomyces peucetius* [85]. Due to their high efficacy, a great number of synthetic analogs have been synthetized [86]. Among these, epirubicin (EPI), which is widely used for the treatment of both carcinomas and sarcomas [87], is synthesized through an epimerization reaction of one hydroxyl group of DOX. One important characteristic of EPI is that, even showing a very similar antitumor activity compared to DOX, it is more glucuronitated and, therefore, better excreted through bile and urine, allowing a safe use at higher doses with respect to DOX [88]. The use of these drugs may often induce acquired resistance [89] and a series of harmful side effects, including mucositis, nausea, vomiting, stomatitis and, mainly, high cardiotoxicity.

To identify the mechanisms of ATC-induced cardiotoxicity, a variety of cellular and molecular pathways have been explored over the last three decades. The precise knowledge of these mechanisms represents an essential prerequisite to identify possible strategies and treatments to inhibit the development of cardiotoxicity. However, despite the great effort, there is still a lack of agreement on which alteration(s) may be the most directly responsible for ATC-induced cardiotoxicity [15]. Given the complexity of this topic, it is not possible to analyze in detail herein all the mechanisms involved in ATC-induced cardiotoxicity. Recently, excellent reviews [15,90] have been published on this topic, and we will hereinafter provide only a brief outline of the mechanisms so far identified (next paragraph). ω-3 PUFA appear as ideal candidates in preventing the development of cardiac events induced by ATC chemotherapy, since these fatty acids are known to induce benefits at a cardiovascular level by positively modulating some of the cellular processes and molecular pathways that, conversely, are harmfully altered by ATC and other chemotherapeutic agents.

### 5.1. Mechanisms of ATC-Induced Toxicity

ATC induce cardiotoxicity with acute, sub-acute or chronic clinical manifestations, and the incidence of chronic cardiac pathologies (mainly cardiomyopathy and congestive heart failure) has been directly related to the amount of drugs accumulated in the heart [91]. Conventionally, the main pathogenic role has been attributed to the oxidative stress generated by an increased production of intracellular ROS, that has been thought to be initiated by the redox cycling of ATC and amplified by the formation of free radicals catalyzed by iron chelated by ATC and, subsequently, accumulated in the heart [91,92]. Moreover, the moderately scarce antioxidant defenses of the myocardium have also been thought to be per se a sufficient reason in explaining the high susceptibility of this tissue to ATC-induced-cytotoxicity [15]. In addition, ATC, and DOX in particular, mainly accumulate in the mitochondria, which are mostly concentrated in the myocardium [93,94]. However, the oxidative stress hypothesis has been challenged by the outcomes of clinical studies showing that antioxidants failed to provide protection against ATC-induced cardiotoxicity [95,96]. Nevertheless, other mechanisms have also been hypothesized. For instance, it has been suggested that cardiotoxicity may also be related to oxidative-independent myocardial cell damage and death associated to the overload of iron in the free form, since DOX can target proteins that specifically bind, transport or regulate the binding and transport of iron [91]. In line with this, it has been shown that the supplementation of rats in vivo with iron produced cardiac free iron accumulation and intensified the cardiotoxic effects of these drugs [97]. On the contrary, iron chelation can prevent ATC-induced cardiotoxicity. Similarly, the iron chelator dexrazoxane has been used to prevent heart pathologies secondary to chemotherapy, not without controversies due to its toxicity and possible carcinogenicity [15]. Other iron chelators have failed to demonstrate the same effectiveness [98,99]. This may be because dexrazoxane has shown to possess additional effects, being able to also inhibit topoisomerase 2β (Top2β) [100], an enzyme that traps DNA and DOX to form a DNA cleavage complex which is able to trigger apoptosis [101]. Several alternative oxidative (i.e., alteration of activity of endothelial nitric oxide synthase (eNOS) and nicotinamide adenine dinucleotide phosphate (NADPH) oxidase) and non-oxidative molecular pathways of ATC-cardiotoxicity have also been suggested, although their detailed description is far beyond the scope of this review [15]. However, it is worth mentioning some mechanisms recently proposed, since ω-3 PUFA can often affect them in an opposite way compared to ATC. Particularly relevant in this context is the inflammatory hypothesis, which considers the activation of the innate immunity and the release of inflammatory cytokines central in the development and progression of the ATC-induced cardiotoxicity [102,103,104]. Similarly, it is worth pointing out the modulatory activity of ATC on the expression of several miRNA involved in the epigenetic regulation of different pathways of apoptosis, since ω-3 fatty acids have also been shown to modulate the epigenetic regulation of gene expression [7,105,106]. 

Furthermore, it is worth noting the emerging hypothesis [90] suggesting a central role for the ATC-induced dysregulation of the autophagic response, since the ability of ω-3 PUFA in regulating the autophagic processes has also been reported [107]. An impaired autophagic process results in an unbalanced cellular proteostasis with an excessive protein load that may ultimately induce cell death. However, although multiple results have shown that DOX deeply affects the autophagic process in the heart, and that, by reversing the DOX effects on autophagy, the cardiac cell damage and death are also reduced, there is still great uncertainty as to whether the DOX-mediated cardiotoxicity may be related to an increase or a decrease in autophagy [90,108,109]. According to the recent analysis of the literature by Bartlett et al. [90], the controversies in this field may be related to the targets examined in the autophagic machinery, and more attention should be focused on the explanation of the “autophagic flux”. An impaired autophagic flux, i.e., a decreased lysosomal degradation of autophagosomes [109,110], could be central in DOX-related cardiotoxicity, and its restoration could reduce the heart damage related to the use of this drug.

Lastly, an important aspect of this subject, recently highlighted by Cappetta et al. [111] is that cardiomyocytes have extensively been considered the main cellular targets of ATC chemotherapy, and cardiomyopathy following the therapy has exclusively been ascribed to their death. However, the vulnerability of other cell types (i.e., cardiac cells: cardiac progenitor cells, cardiac fibroblasts, vascular cells; non cardiac cells, such as bone marrow cells and endothelial progenitor cells) to DOX has also been recently described, and involved in the DOX-mediated cardiotoxicity [111]. Thus, these cell types should also be taken into account for further research on ATC-induced cardiotoxicity.

### 5.2. Available Evidence for ω-3 Prevention of ATC-Mediated Cardiotoxicity

Only a small number of preclinical studies have hitherto investigated the effect of ω-3 PUFA on the ATC-induced cardiotoxicity, and their results are quite inconsistent. Among the in vivo animal studies analyzed (Table 1), only two [112,113] reported functional alterations in the hearts from animals orally supplemented with ω-3 PUFA and subject to DOX treatments. On the other hand, two studies [114,115] did not find any effect on cardiac morphology/functions, one [81] reported mixed effects (i.e., both no effects on some parameters and positive effects on others), and the remaining studies supported potential preventive effects of these fatty acids [78,79,116,117]. Moreover, one in vivo study [118] (Table 1) did not investigate the functional/morphological effect on the heart, but reported the preventive effects that these fatty acids induced at biochemical and molecular levels in this area. Such beneficial effects were also reported by all the four in vitro studies [80,82,83,119] (Table 2) conducted in cardiomyocytes treated with ω-3 PUFA and DOX.

The great variability in the experimental conditions used in the animal studies may explain the inconsistency of their outcomes. For instance, various cumulative doses and ways of administration of ATC have been used which can result in different concentrations of this drug at the cardiac level. Generally, these drugs are administered to humans through intravenous (IV) injections via a central line or a peripheral venous line. In three of the works analyzed, ATC were administered intravenously [81], or “intracoronary” [113,120], whereas most of the studies were performed by injecting the drug intraperitoneally (IP) [78,79,114,117], and the remaining reports did not specify the route of administration. The intracoronary DOX administration was performed in an ovine model, where these vessels are large and easily reached. Possibly, this way of inoculation was used to stress the experimental conditions, in order to obtain higher cardiac DOX concentrations and, consequently, better detect alterations in the heart morphology and functions. Interestingly, one of these studies (Table 1), performed in the ovine model [113], reported a negative effect (in terms of dilation of the left ventricle and greater decline in ejection fraction) of an oral supplementation (19 weeks) with 23 mL of Omega 18/12 fish oil (FO) (Melrose Health, Mitcham, Victoria, Australia, containing 30% EPA + DHA) given before, simultaneously, and after the DOX treatment (cumulative DOX dose: 3–4 mg/kg body weight). However, the same group of authors [120] had previously used the same ovine model, and had found protective effects at the atrial level (suppression of left atrial dilation and interstitial fibrosis, as well as decreased alteration in atrial conduction) (Table 1). In this case, the sheep were supplemented daily with 10 mL of the same FO, and injected with comparable amounts of DOX (cumulative DOX dose: 3.6 mg/kg body weight). Thus, it is not clear if the contrasting effects between the two works could be ascribed to the different cardiac regions and parameters examined or to the different supplementation timing of a quantitatively comparable (3 g/day) EPA + DHA supplementation. The only other work reporting negative health effects from ω-3 PUFA (increased mortality and worst cardiac performance, i.e., reduction of left ventricular fractional shortening, LVFS) was conducted by Matsui et al. [112] in rats (Table 1). In this case, the animals were dietary supplemented with 10% menhaden oil (MO, Sigma, St. Louis, MO, USA, containing approximately 20–30% EPA + DHA, for seven weeks in total). This supplementation provided the rats with approximately 2.0–3.0 g EPA + DHA/kg body weight/day (see [121] for calculations). The animals received three weeks of MO treatment, and were then injected with DOX (cumulative dose: 15 mg/kg body weight, injection and timing not specified). On the contrary, in the two studies conducted by Germain et al. [114,115], where ω-3 supplementations were found neutral for heart health, rats were treated daily with epirubicin (EPI, cumulative dose: 9–15 mg/kg), and their diet was enriched with 15% sardine oil (SO, Polaris Biotechnique, Columbus, OH, USA, containing about 30% EPA + DHA, and corresponding to a dose of about 4.5 g EPA + DHA/kg body weight) or with 15% algal-derived triglyceride DHASCO oil (containing about 40% DHA and corresponding to a dose of about 6.0 g DHA/kg body weight) [115] (Table 1). These authors also added anti-or pro-oxidants to the diets, but never found changes at the cardiac levels, either at the lowest dose or the highest dose of ω-3 [115]. It should be emphasized that, compared to the work by Matsui et al. [112], in which ω-3 induced harmful cardiac effects in rats treated with DOX, in the studies by Germain et al. [114,115], higher doses of EPA + DHA were administered to the rats (4.5–6 g/kg body weight vs. 2–3 g/kg body weight). This is quite unusual, since, if ω-3 had the potential to increase the ATC-induced cardiotoxicity, we would expect to observe greater alterations at the cardiac level by treating the animals with higher doses of these fatty acids. However, it should be underlined that Germain et al. [114] did not use DOX, but EPI, which, at comparable cumulative doses, is less cardiotoxic than DOX [122]. It is also interesting to notice that the neutral effects obtained by Germain et al. [114,115] were interpreted by the authors as a favorable result, since their studies were aimed at assessing whether ω-3 PUFA would exacerbate the CV effect induced by these drugs, in addition to chemo-sensitizing breast cancer to ATC therapy.

The beneficial effects on functional parameters at the cardiac level observed by Teng et al. [78] (Table 1) were obtained by supplementing rats with 6 g FO/kg body weight by gastric gavage. The type of FO was not specified, but, if it had a EPA + DHA content of approximately 20–30%, as it is generally observed in the FO mostly used in animal studies (menhaden oil or MaxEPA, see [121]), the rats would have received a daily dose of about 1.5 g EPA + DHA, that is about a half of that ingested by the rats showing cardiotoxic effects in the work by Matsui et al. [112]. Moreover, it should be pointed out that these authors [112] supplemented ω-3 PUFA incorporated in the diet and not, as in this work, by gastric gavage [78] that, presumably, allows a more precise intake and a higher bioavailability of these fatty acids in the serum and tissues. Perhaps, also the longer period of ω-3 supplementation (eight weeks vs. four weeks) used in this case [78] allowed to obtain healthy cardiac effects, although this treatment was performed after the DOX treatment (IP injection) and not before it, as in the study by in Matsui et al. [112] (where the type of DOX injection was not specified). More recently, Uygur et al. [79] (Table 1) also obtained protective effects at the cardiac levels (in terms of improved cardiac histological parameters and reduced apoptotic index) by administering the rats by gavage 0.4 g/kg body weight of EFA capsules (New Life EFA S-1200, Eurocaps Limited, Dukestown, Tredegar, UK, containing 60% EPA + DHA), corresponding to a dose of ω-3 (0.240 g/kg body weight) even lower than that of Teng et al. [78]. Similar to the study by Matsui et al. [112], in this case, this very low dose was given for four weeks before the treatment with DOX, that, however, was administered at a cumulative dose twice what Matsui et al. [112] had administered the animals (30 mg/kg vs. 15 mg/kg). Overall, the works analyzed to date suggest that, providing ω-3 at relatively lower doses (lower than about 2.0 g/kg body weight in rats) in the presence of ATC treatments, may induce more favorable cardiac effects. Moreover, they suggest that, even extremely low doses of ω-3 may be efficient, provided that the treatment is performed before ATC, even if the drug is administered at high cumulative doses.

Healthy cardiac effects were also obtained in other works using models that differed for many aspects from those so far analyzed. In one of them, Xue et al. [81] (Table 1) administered EPA and DHA (0.19 g/kg body weight EPA and 0.18 g/kg body weight DHA) every other day in a parenteral solution via tail vein of anesthetized rats bearing syngeneic mammary adenocarcinoma xenografts. This treatment was performed one week before and seven weeks after the DOX treatment with a cumulative dose of 6 mg/kg. Despite the difficulty in comparing this model to the others so far analyzed, it is interesting since it reflects the possible supplementations with ω-3 in oncological patients undergoing ATC therapy. In this case, mixed effects were obtained regarding the parenteral infusion with EPA and DHA. Neutral effects were observed for some parameters, such as the DOX-induced increase in the left ventricular end-diastolic and end-systolic dimensions (LVEDD and LVESD), DOX-induced lipid peroxidation, antioxidant factors and apoptosis in the myocardium. Among the positive effects, there were the prevention of the DOX-induced increase in the plasma cardiac troponin I (cTNI) levels, and a trend for better preserved left ventricular function (left ventricular fractional shortening, LVES, and left ventricular ejection fraction, LVEF) and morphology (suppressing DOX-induced LV dilation). The in vivo work by Schjøtt et al. [116] is difficult to compare with other in vivo studies. It was performed by supplementing (for two weeks) rats by gavage with very low daily doses of EPA or DHA-ethyl esters (EPA or DHA, approximately 0.03–0.045 g/kg body weight, suspended in carboxymethylcellulose), and their effect was evaluated on the hearts isolated and perfused with ex vivo DOX. Although the experimental conditions were so peculiar, it is particularly worth noting that extremely low doses of EPA or DHA resulted to be protective (by decreasing the aortic pressure) on the hearts challenged ex vivo with DOX. In another in vivo work, Yu et al. [117] (Table 1) investigated the effect of α-linolenic acid (ALA, 18:3ω-3), the essential fatty acid precursor of EPA and DHA. Although ALA is known to be scarcely converted in vivo into the longer chain PUFA, EPA and DHA [123], it should be taken into account that experiments performed in rats have demonstrated that different tissues possess different abilities to endogenously synthesize long-chain ω-3 PUFA from ALA. In particular, the heart is one of the sites displaying the highest level of endogenous synthesis of long-chain ω-3 PUFA from ALA [118]. A very low dose of ALA (0.05 g/kg body weight) was administered by gavage to rats three days before and a week after the DOX treatment (cumulative dose: 17.5 mg/kg) [117]. The ALA treatment prevented the alterations induced by DOX on numerous morphologic and functional parameters at the cardiac level. Moreover, these results agree in suggesting that relatively low doses of ω-3 PUFA may be more effective in preventing chemotherapy-induced side-effects in the heart. It is worth pointing out that, in this case, ALA supplementation decreased the DOX-induced oxidative stress, confirming the result observed in the study by Uygur et al. [79], where (see above) low doses of EFA capsules (at high levels of EPA and DHA, but also containing ALA) were administered. On the contrary, the parenteral solution infused by Xue et al. [81], and containing comparable amounts of EPA + DHA, did not affect the other several investigated parameters of oxidative stress. On the other hand, lipoperoxidation (LP) was found increased in the heart tissue and the level of vitamin E decreased by Matsui et al. [112], and these modifications were included by the authors among the harmful effects exerted by these fatty acids at cardiovascular levels. Interestingly, in a work [118] (Table 1) where rats were subject to chemically-induced mammary carcinogenesis, a supplementation with doses of DHASCO oil furnishing approximately 3.5 g DHA intake/kg body weight [121,124], prior and during the EPI treatment, did not induce any increase in LP in normal tissue, including the cardiac tissue. Meanwhile, the cardiac total antioxidant activity, as well as that of the antioxidant enzymes SOD and GPx was found to be increased. On the contrary, LP increased remarkably in the tumor tissue, due to the lack of adjustment of the antioxidant reserve, which may potentially improve the outcome of ATC chemotherapy. Moreover, two in vitro investigations (Table 2) [80,82] found that EPA and DHA added to the culture medium had the potential to suppress the DOX-induced ROS production of H9C2 cardiomyoblast cell line. It was also found that the treatment of cardiomyoblasts in vitro with DHA [82] or sardine oil embedded in vanillic acid-chitosan microparticles [83] could also suppress the DOX-induced expression/activation of NF-κB [82,83], a transcription factor activated by the increase of ROS at the cellular level, and, furthermore, involved in the transcription of inflammatory cytokines. Compatibly, DHA was also found to suppress [82] the DOX-induced expression of TNF-α, IL-6, MCP-1 and IL-1β. In the same work [82] DHA also reduced the DOX-mediated expression of the enzyme iNOS, related to the generation of the powerful oxidant peroxynitrite from NO. Some of these in vitro studies (Table 2) reported the contrasting effect exerted by ω-3 treatment on phenomena related to the DOX-induced apoptosis, i.e., the decrease of the mitochondrial membrane potential, and caspase-3 activation [80,83]. Lastly, one of these studies reported the ω-3 ability to suppress the increase in (Ca^2+^)_i_ related to the alteration induced by DOX in sarcoplasmic reticulum Ca^2+^ release [119].

## 6. Conclusions

Overall, the extreme variability in the experimental in vivo models makes their analysis and comparison very difficult. However, from the experiments performed in rats, it is possible to conclude that the level of ω-3 PUFA intake represents one of the main factors determining whether they exert protection against ATC-induced cardiotoxicity. We have observed that relatively low doses administered to rats with the diet (0.5 g/kg body weight for ALA, and ranging from 0.2 to 1.5 g/kg body weight for EPA + DHA or DHA alone) could prevent the development and progression of DOX-mediated cardiotoxicity in vivo. Even lower doses of ω-3 PUFA given to rats in vivo (0.030–0.045 g/kg body weight) resulted in cardioprotective effects in hearts excised and perfused ex vivo with DOX. On the contrary, higher doses (2.0–3.0 g ω-3/kg body weight) exacerbated the harmful effects of DOX at the cardiac level. Interestingly, however, even higher doses of EPA and/or DHA (4.5 g EPA + DHA/kg body weight or 6.0 g DHA/kg/body weight) appeared neutral, not adding further risks to the cardiotoxicity if associated to a treatment with EPI, an ATC showing less cardiotoxicity than DOX. Thus, these outcomes suggest that, to obtain a protective effect, relatively low doses of ω-3 PUFA should be administered (lower than 2.0 g/body weight in rats). These doses would probably not be sufficient to induce cytotoxicity driven by lipoperoxidation and high level of oxidative stress that, conversely, are known to be induced by high concentrations of these fatty acids [125]. On the contrary, as demonstrated by the outcomes of both the in vivo and in vitro studies, low and protective doses may not alter or may even decrease the ATC-induced lipoperoxidation. They may also not affect or even reduce the level of ATC-induced ROS formation, and, lastly, may not modify the ATC-induced decrease in antioxidant enzyme expressions or activities, or even induce them. 

**Figure 1:**
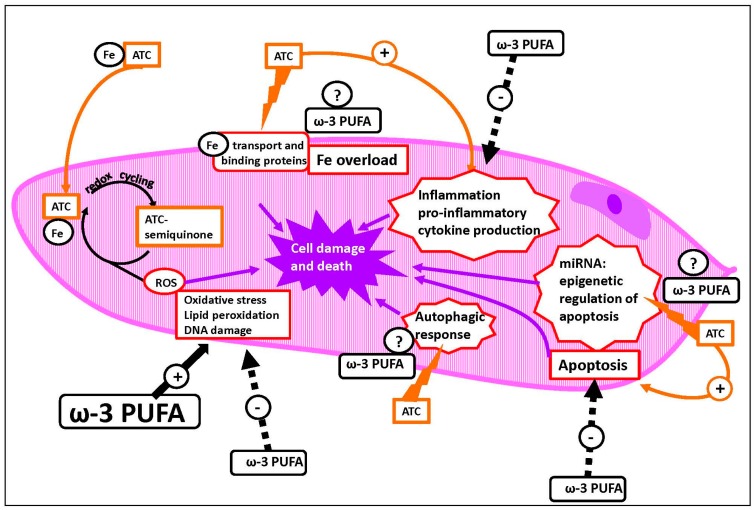
Potential protective effects of ω-3 polyunsaturated fatty acids (PUFA) against anthracyclines (ATC)-induced cardiotoxicity. Diagram of a cardiomyocyte and some mechanisms of ATC-induced cardiotoxicity that were found to be either activated or inhibited by ω-3 PUFA (ω-3 PUFA) (indicated by plain black arrows with + sign or dashed black arrows with – sign, respectively), or that could be potentially affected by these fatty acids (see in the text for more details). The question marks indicate some mechanisms that we have suggested, and through which ω-3 PUFA could potentially prevent ATC-induced cardiotoxicity toxicity (see in the text for more details). Orange arrows indicate mechanisms of ATC-induced cardiotoxicity. Purple arrows indicate pathways leading to cell death.

Moreover, in a future prospective, it should be emphasized that, although the mechanisms of cytotoxicity related to the oxidative stress are those mainly investigated by the ATC/ω-3 PUFA combined studies, recently, several other important and more specific mechanisms have been sought to explain the health effects of ω-3 PUFA, both in general and at CV levels (Figure 1). Some of them involve their anti-inflammatory activities, as well as their ability to negatively modulate some molecular pathways, such as those leading to the activation of NF-κB or those involved in autophagy [126,127,128,129], or to influence the epigenetic regulation [7,130]. Interestingly, these mechanisms represent some of those more recently involved in the ATC-induced cardiotoxicity. Since they often appear to be modulated by ATC and ω-3 PUFA in an opposite way, it would be worthwhile to investigate their involvement in future combined studies examining the effects ω-3 PUFA and ATC at CV level. Remarkably, it has been recently observed that one of the consequences of iron overload at the hepatic level is the inhibition of the Δ-5 and Δ-6 desaturases, which prevents the endogenous synthesis of LC-ω-3 PUFA, and leads to their depletion with harmful consequences for the cells [131]. Since iron overload is also considered one of the main mechanisms of ATC-induced cardiotoxicity, it would be stimulating to investigate whether it may suppress the synthesis and the level of LC-ω-3 PUFA also at cardiac levels, and if the impaired synthesis could be related to ATC-cardiotoxicity.

## Figures and Tables

**Table 1:** Effect of in vivo ω-3 PUFA treatments on ATC-induced cardiotoxicity in animal models.

Experimental Model	ATC Treatment	ω-3 PUFA Treatment	Treatment Combined with ω-3 PUFA	Control Condition (Alternative to ω-3 PUFA or/and ATC Treatments)	ω-3 PUFA-Induced Morphological/Functional Effects at CV Level	ω-3 PUFA-Induced Biochemical and Molecular Effects	Ref.
Rats bearing NMNU-induced mammary tumors	EPI (3 mg/kg/week; IP) for 3 weeks	Diet containing 15% SO (6 weeks)	±LP inducers * in drinking water or ± vitamin E (100 IU/kg diet)	none	**NO EFFECTS** SO ± LP inducers * or SO ± vit E: no variation in EPI-induced increase in LVEDP in almost 10% of rats in all groups	n.d.	[114]
Hearts isolated from rats supplemented with ω-3 PUFA and perfused ex vivo with EPI	EPI heart perfusion ex vivo: 0.2 mg/min/10 min	1.0 mL (300 mg) EPA or DHA ethylesters suspended in 0.5% carboxymethylcellulose by gavage (1st week); 1.5 mL (450 mg) (2nd week)	none	1.0 mL (1st week) or 1.5 mL (2nd week) olive oil (alternative to EPA or DHA treatments)	**PROTECTIVE EFFECTS** Heart from EPA- or DHA-treated rats perfused with EPI: lower aortic pressure (index of coronary resistance) than in olive oil-treated hearts	No difference in the heart release of LDH among groups during and after EPI infusion	[116]
Sprague-Dawley rats	DOX (cumulative dose): 15 mg/kg after 4 weeks of FO treatments	Diet containing 10% MO (4 weeks prior and 3 weeks after DOX treatment)	none	0.28 M dextrose solution (alternative to DOX treatment)	**HARMFUL EFFECTS** FO diet: -highest mortality; -further reduction in cardiac LVFS	FO diet: increase in myocardial LP and decrease in vit E level	[112]
Sprague–Dawley rats	After oil treatments: EPI (weekly, cumulative doses): 9 mg/kg (exp. 1) 15 mg/kg (exp. 2)	Diet containing 15% SO or DHASCO oil (% not reported) (at least 3 weeks before EPI treatment)	±LP inducers in drinking water or vitamin E (100 IU/kg diet)	Palm oil (alternative to DHASCO oil treatment)	**NO EFFECTS** SO or DHASCO oils (± anti- or pro-oxidants): no changes in EPI-induced: -mortality; -alterations of LVEDP and LVSP and left ventricular systolic pressure; -histological damages	n.d.	[115]
Male Sprague-Dawley rats	IP DOX injection (2 mg/kg/week) (8 weeks)	FO ^§^ (0.6% of body weight) daily, by gavage (for 8 weeks, after DOX treatment)	none	0.9% normal saline (alternative to FO)	**PROTECTIVE EFFECTS** FO supplementation: -Lower LVEDD and LVESD -Higher LVEF and LVFS	n.d.	[78]
Merino wether sheep	Intracoronary DOX infusions (1.0 mg/kg, every other week) (for 3–4 weeks)	Oral supplementation: 1.8 g EPA + 1.2 g DHA/day ° (1 week prior and 13–15 weeks after the last DOX infusion)	none	No supplementations, sham operated; (Alternative to ω-3 and DOX treatments) Olive oil (10 mL)	**PROTECTIVE EFFECTS** EPA + DHA supplementation, suppression of DOX-induced: -left atrial dilation and interstitial fibrosis; -alterations in atrial conduction	n.d.	[120]
Merino wether sheep	Intracoronary DOX infusion (1.2 mg/kg, every other week) (for 3 weeks)	Oral supplementation: ω 18/12 FO (23 mL) (3 times/week for 3 weeks prior and 16 weeks during and post DOX treatment	none	Oral supplementation with 23 mL olive oil (alternative to FO)	**HARMFUL EFFECTS** FO supplementation: left ventricular dilatation; greater decline in ejection fraction	More frequent elevation of serum troponin-T after DOX treatment in ω-3 treated sheep	[113]
Female Sprague-Dawley rats	IV EPI injection (0.8 mg/kg once a week) (for 6 weeks)	Oral supplementation DHASCO oil 80 g/kg diet (45% DHA) for 12–13 weeks prior and 6 weeks during EPI treatment	none	Palm oil-based diet	n.d.	In cardiac tissue: no changes in LP and total antioxidant activity; increased antioxidant enzyme (GPx, SOD) activity	[118]
Male Sprague-Dawley rats	DOX (2.5 mg/kg, IP) (from the 4th day, every other day) (for 7 times)	Pretreatment with ALA (500 µg/kg body weight) by gavage (3 days); from the 4th day: every other day (for 7 days)	none	Oral supplementation with normal saline throughout the experiments (alternative to ALA and DOX treatments)	**PROTECTIVE EFFECTS** ALA supplementation, suppression of DOX-induced: -cardiac histopathological alterations, -reduction of LVEDV, SV, and EF, -increase in HW/BW, -cardiomyocyte apoptosis	ALA prevented DOX-induced: -in serum: increase in BNP, CK-MB, LDH, cTnI levels; -in cardiac tissue: LP increase and antioxidant enzymes ** decrease; -caspase-3 activation and changed BAX and BCL2 expression; -p-AKT and p-ERK decreased expression	[117]
Sprague-Dawley rats	Single IP DOX dose (30 mg/kg, after 30 day ω-3 PUFA treatment)	Pretreatment with ω-3 PUFA capsules (New Life EFA S-1200 (400 mg/kg/d, by gavage) (30 days before DOX injections)	none	0.4 mL/kg saline by gavage (alternative to ω-3 PUFA and DOX treatments)	**PROTECTIVE EFFECTS** PUFA treatment: -improved cardiac histological appearance; -reduction of apoptotic index (Tunel-positive cardiomyocytes)	Decrease in MDA levels; increase in SOD and GPx activities	[79]
Female Fisher 344 rats bearing syngeneic MatBIII mammary adenocarcinoma xenograft	DOX (1 mg/kg, IV) starting as tumor mass =1.2 cm^3^ (for 6 days and then weekly for 6 weeks)	Parenteral solution (tail vein) containing 0.19 g/kg EPA + 0.18 g/kg DHA) every other day (6 days before DOX treatment until day 50)	±Parenteral solution containing glutamine (0.35 g/kg) ± EPA + DHA	Parenteral saline (alternative to ω-3 PUFA, DOX)	**MIXED EFFECTS** EPA + DHA solution: -No modifications in DOX-induced: increase in LVEDD, LVESD and apoptosis; -Partially improved LV dilation and function (no statistical significance); -Reversion of glutamine positive cardiovascular effects	EPA + DHA parenteral solution prevents DOX-induced elevation of plasma cTnI levels; No effect on DOX-induced lipid peroxidation and enzymatic and non-enzymatic antioxidants in cardiac tissue	[81]

AKT, protein kinase B; ATC, Anthracyclines; BAX, bcl-2-like protein 4, BCL-2, B-cell lymphoma 2; BNP, brain natriuretic peptide; CAT, catalase; CK-MB, creatine kinase-MB; cTnI, cardiac troponin I; CV, cardiovascular; DHASCO oil, algal-derived triglyceride containing 40% DHA; DOX, Doxorubicin; EF, ejection fraction; EPI, Epirubicin; ERK, extracellular signal–regulated kinase; FO: Fish oil; GPx, Glutathione peroxidase; HW/BW, heart weight/body weight; IP: Intraperitoneal; IV, Intravenous; LP, lipid peroxidation; LDH, lactate dehydrogenase; LVEDD, left ventricular end-diastolic dimension; LVEDP: left ventricular end diastolic pressure; LVEF: left ventricular ejection fraction; LVESD, left ventricular end-systolic dimension; LVFS: left ventricular fractional shortening; LVSP, left ventricular systolic pressure; MDA, malondialdehyde; MO: menhaden oil; NMNU: *N*-methyl nitrosourea; n.d.: not determined; SO: sardine oil; SOD, Superoxide dismutase; SV: stroke volume; vit. E, vitamin E. *: LP inducers: 20 mg/day dehydroascorbate + 0.2 mg/day menadione. ^§^: FO, Fish oil not specified; **: SOD, GPx and CAT; °: equivalent to a daily intake of 10 mL FO, i.e., 3 g long-chain ω-PUFA.

**Table 2:** Effect of ω-3 PUFA treatments on ATC-induced alterations in cardiomyocytes in vitro.

Experimental Model	ATC Treatment	ω-3 PUFA Treatment	Additional Treatments	Control Condition	ω-3 PUFA Effect on DOX-Induced Cardiac Cell Viability	Effects of ω-3 PUFA in Combination with DOX at Biochemical and Molecular Levels	Ref.
Isolated adult rat cardiomyocytes perfused with CaCl2 Krebs solution	100 µM DOX (after 20 min DHA treatment)	Pre-treatment with 10 µM DHA (20 min)	none	No treatment with DHA ± treatment with DOX	n.d.	**PROTECTIVE** DHA pretreatment: Inhibition of DOX-induced (Ca^2+^)_i_ increase	[119]
H9C2 cardiomyoblast cell line	1 μM DOX in DMEM-10% FBS (24 h) after 24 h EPA/DHA treatment	Pre-treatment with 100 μM EPA or 50 μM DHA in DMEM-0.1% BSA (24 h)	none	No treatment with ω-3 PUFA ± treatment with DOX	n.d.	**PROTECTIVE** EPA or DHA pretreatment: prevention of DOX-induced: -decrease in UCP2 levels -increase in ROS production -MMP decrease	[80]
H9C2 cardiomyoblast cell line	5 μM DOX in DMEM-10% FBS (4 h)	Co-treatment with 10 μM DHA-FFA (4 h)	none	No treatment with DHA ± treatment with DOX	Increased viability	**PROTECTIVE** DHA co-treatment: suppression of DOX-induced: -ROS production; -expression of TNF-α, IL-6, MCP-1, iNOS, and IL-1β; -phosphorylation of IκB-αand NF-κB/P65	[82]
H9C2 cardiomyoblast cell line	20 mM DOX in DMEM-10% FBS (1 h)	After DOX treatment: 1.25 mg/mL SO-loaded Va-g-Ch microparticles (SO-M)	none	No treatments	n.d. *	**PROTECTIVE** SO-M treatment: suppression of DOX-induced: -caspase-3 activation; -increased expression of NF-κB	[83]

ATC, Antracyclines; BSA, bovine serum albumin; DHA-FFA, DHA bound free fatty acid; DMEM, Dulbecco modified minimum essential medium; DOX, doxorubicin; IL, interleukin; MCP-1, monocyte chemoattractant protein 1; MMP, mitochondrial membrane potential; n.d., not determined; SO, sardine oil; SO-M, sardine oil-loaded microparticles; TNF-α, tumor necrosis factor α; UCP2: uncoupling protein 2; Va-g-Ch: vanillic acid-grafted chitosan; * Higher cardiomyocyte viability, lower oxygen reactive species (ROS) production, and MMP near to normal level, evaluated only with SO-M treatment alone, compared to DOX-treatment alone.
